# Supplementary material for: The causal role of circulating inflammatory markers in osteoporosis: a bidirectional Mendelian randomized study
Source: Front Immunol. 2024 Jul 18;15:1412298. doi: 10.3389/fimmu.2024.1412298 (PMC11291241; doi:10.3389/fimmu.2024.1412298)
Supplement: Supplementary file 1 [file DataSheet_1.zip › supplementary material/OSTEOPOROSIS-CIM/GCST90274826.tsv.gz_forest.pdf]

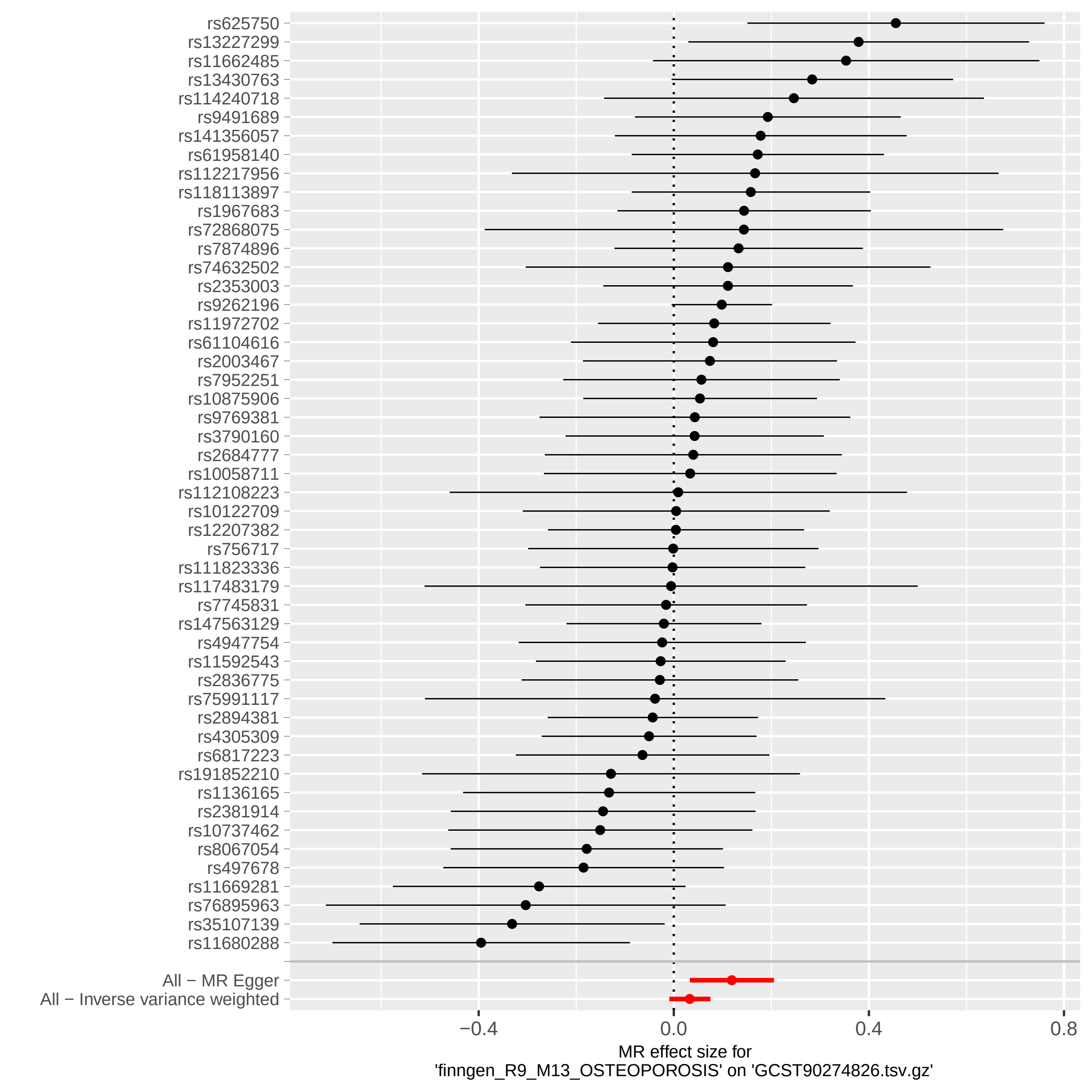
rs625750
rs13227299
rs11662485
rs13430763
rs114240718
rs9491689
rs141356057
rs61958140
rs112217956
rs118113897
rs1967683
rs72868075
rs7874896
rs74632502
rs2353003
rs9262196
rs11972702
rs61104616
rs2003467
rs7952251
rs10875906
rs9769381
rs3790160
rs2684777
rs10058711
rs112108223
rs10122709
rs12207382
rs756717
rs111823336
rs117483179
rs7745831
rs147563129
rs4947754
rs11592543
rs2836775
rs75991117
rs2894381
rs4305309
rs6817223
rs191852210
rs1136165
rs2381914
rs10737462
rs8067054
rs497678
rs11669281
rs76895963
rs35107139
rs11680288
All − MR Egger
All − Inverse variance weighted
−0.4
0.0
0.4
0.8
MR effect size for
'finngen_R9_M13_OSTEOPOROSIS' on 'GCST90274826.tsv.gz'
